# Prevention of anxiety disorders in primary care: A feasibility study

**DOI:** 10.1186/1471-244X-12-206

**Published:** 2012-11-22

**Authors:** Neeltje M Batelaan, Jan H Smit, Pim Cuijpers, Harm WJ van Marwijk, Berend Terluin, Anton JLM van Balkom

**Affiliations:** 1 Department of Psychiatry, VU University Medical Center and GGZ inGeest, AJ Ernststraat 1187, Amsterdam, 1081 HL, The Netherlands; 2 EMGO-institute, VU University Medical Center, Amsterdam, The Netherlands; 3 Department of Clinical Psychology, VU University, Amsterdam, The Netherlands; 4 Department of General Practice, VU University Medical Center, Amsterdam, The Netherlands

**Keywords:** Anxiety disorders, Prevention, Public Mental Health, Screening

## Abstract

**Background:**

Anxiety disorders are highly prevalent in primary care and cause a substantial burden of disease. Screening on risk status, followed by preventive interventions in those at risk may prevent the onset of anxiety disorders, and thereby reduce the disease burden. The willingness to participate in screening and interventions is crucial for the scope of preventive strategies, but unknown. This feasibility study, therefore, investigated participation rates of screening and preventive services for anxiety disorders in primary care, and explored reasons to refrain from screening.

**Methods:**

In three general practices, screening was offered to individuals visiting their general practitioner (total n = 2454). To assess risk status, a 10-item questionnaire was followed by a telephone interview (including the CIDI) when scoring above a predefined threshold. Preventive services were offered to those at risk. Participation rates for screening and preventive services for anxiety disorders were assessed. Those not willing to be screened were asked for their main reason to refrain from screening.

**Results:**

Of all individuals, 17.3% participated in initial screening, and of those with a possible risk status, 56.0% continued screening. In 30.1% of those assessed, a risk status to develop an anxiety disorder was verified. Of these, 22.6% already received some form of mental health treatment and 38.7% of them agreed to participate in a preventive intervention and were referred. The most frequently mentioned reasons to refrain from screening were the emotional burden associated with elevated risk status, the assumption not to be at risk, and a lack of motivation to act upon an elevated risk status by using preventive services.

**Conclusions:**

Screening in general practice, followed by offering services to prevent anxiety disorders in those at risk did not appear to be a feasible strategy due to low participation rates. To enable the development of feasible and cost-effective preventive strategies, exploring the reasons of low participation rates, considering involving general practitioners in preventive strategies, and looking at preventive strategies in somatic health care with proven feasibility may be helpful.

## Background

Anxiety disorders are prevalent, often have a chronic course, and are associated with functional limitations. Given these features, preventing the development of anxiety disorders seems a good strategy. Hence, screening of individuals on risk factors and early stages of the disorder offers the opportunity to provide preventive interventions, and thereby limit the disease burden for both the individual and the society.

Some decades ago, Wilson and Jungner identified criteria defining situations in which screening on risk factors and early stages of the disorder is appropriate [1](Table 1). Based on these criteria, screening on risk factors and subclinical anxiety is indeed warranted. First, anxiety disorders are one of the most common psychiatric disorders with a lifetime prevalence of approximately 20% [2-4] and an annual incidence rate of 3% among adults [5]. They are regarded an important health problem [4,6] given their often chronic course [7] and functional limitations [6,8,9]. Related to their chronic course, comorbid disorders often occur [10], thereby negatively impacting on the outcome. Second, effective preventive interventions based on cognitive behavioural treatments are available [11-16]. It is plausible that these interventions are acceptable for those at risk because interventions are not associated with any hazard and can be provided according to the individuals’ preference, i.e. in an individual format, a group intervention or a format using the internet. Third, screening implies that risk factors for anxiety disorders are known and can be assessed accurately. Previously, we defined target groups for the prevention of anxiety disorders, in which the largest public health benefit for the lowest effort can be achieved [17]. These target groups consist of individuals 1) with a (subthreshold) panic attack in the past year, 2) with an affective disorder in the past year, or 3) with a history of anxiety disorders combined with low mastery. These target group characteristics can be assessed relatively simple and without any risks involved, i.e. by a self report questionnaire and a telephone interview. Given the substantial costs associated with anxiety disorders [18], case-finding and subsequent treatment may well be cost-effective.

**Table 1:** **Assessment criteria for screening**[1]

	
1	The condition sought should be an important health problem.
2	There should be an accepted treatment for patients with recognized disease.
3	Facilities for diagnosis and treatment should be available.
4	There should be a recognizable latent or early symptomatic stage.
5	There should be a suitable test or examination.
6	The test should be acceptable to the population.
7	The natural history of the condition, including development from latent to declared disease, should be adequately understood.
8	There should be an agreed policy on whom to treat as patients.
9	The cost of case-finding (including diagnosis and treatment of patients diagnosed) should be economically balanced in relation to possible expenditure on medical care as a whole.
10	Case-finding should be a continuing process and not a “once and for all” project.

Given the assessment criteria, screening on high risk groups for the development of anxiety disorders is thus both appropriate and possible. However, the level of willingness of primary care patients to participate in screening and in interventions to prevent anxiety disorders is unknown. It is imaginable that some individuals refrain from screening because the knowledge of being at risk may be emotionally stressful. Likewise, using preventive services implicates not only time investment, but also a continuous confrontation with being at risk. In addition, in individuals visiting primary care, prevention of anxiety disorders may not be on their (somatic) agenda. Participation in screening and preventive interventions is crucial with regard to the population health benefit that can be achieved by preventive strategies, and with regard to the cost-effectivity of such strategies. Given the importance of participation rates for large scale implementation of preventive strategies, we conducted a study assessing the willingness of individuals for screening and preventive interventions in primary care.

In three general practices, screening was offered to patients visiting their general practitioner (irrespective of the reason for their visit). When risk status appeared present, a preventive intervention of their choice was offered. We investigated the proportion of individuals willing to be screened, and the proportion willing to accept a preventive intervention in those at risk.

## Methods

In 2010 and 2011, we consecutively offered screening in three general practices located in Amsterdam, Leiden and Almere, thereby covering different regional parts of the Netherlands. Screening was offered free of charge to any individual between 18 and 65 years old who spoke sufficiently Dutch when visiting their general practitioner, irrespective of the reason of their visit. Trained research assistants offered a package to all visitors, including an information letter, the question whether they were willing to be screened for their risk status to develop an anxiety disorder in the oncoming year, a few questions regarding socio-demographics, and the initial screening questions. Individuals could either fill in the questions directly while waiting, or could fill them in at home and return the package in a postage-paid envelope.

The information letter included information about the frequent occurrence of anxiety disorders, the availability of effective preventive interventions, the possibility to assess their risk status, and the procedure.

The research proposal has been approved by the Ethical Review Board of VU-University Medical Center. The Ethical Review Board concluded that, given that the research used fully disidentified data only, informed consent was not required. The information letter provided to all potential participants described that disidentified data would be used to assess the willingness for screening and interventions to prevent anxiety disorders.

If patients refrained from screening, they were asked to provide several socio-demographic characteristics (gender, age, education), and were asked to report the reason of their refusal. If patients agreed to be screened, questions regarding social-demographics were followed by 10 screening questions. These aimed to identify those high-risk groups for the development of anxiety disorders we mentioned in the introduction: individuals 1) with a (subthreshold) panic attack in the past year, 2) with an affective disorder in the past year, or 3) with a history of anxiety disorders combined with low mastery [17]. To screen for panic attacks and affective disorder, we used screening questions regarding panic and depressive mood from the Web-based Screening Questionnaire (WSQ;[19]). The WSQ is a questionnaire screening for the presence of common mental disorders. Symptoms during the past year were included. Validated cut-off scores of the WSQ were used. To screen for anxiety disorders in the past, two questions were formulated. The first asked for anxiety problems in the past, the second whether these anxiety problems had resulted in limited functioning or suffering or had resulted in seeking treatment. Finally, mastery was assessed using an adapted version of the Mastery Scale [20], including 5 items. The total score provides information about the degree of control one assumes to have over his life. In correspondence with previous research [17,21], low mastery was defined as a score lower than or equal to 18. A possible high risk status was regarded present if individuals reported positively on 1) the screening question of panic attacks, or 2) the screening question on depressive mood, or 3) on the questions regarding anxiety problems in the past that had resulted in limited functioning or suffering or for which treatment was indicated, combined with low mastery.

If a high risk status was absent, individuals were informed per email or per post (depending on their preference). It was thereby mentioned that the future onset of an anxiety disorder could not be ruled out based on a screening. Individuals scoring positively on the initial screening questions were regarded as having a *possible* high risk status because the screening questions provide insufficient assurance of a proper selection of people at high risk for two reasons. First, based on a screening question the presence of a fully developed anxiety disorder cannot be ruled out. Second, there is a risk of false positives because the WSQ is over inclusive [19].

In individuals with a possible high risk status, initial screening questions were therefore followed by a telephone interview. These individuals were, therefore contacted within two weeks. If necessary, individuals were contacted at different times a day up till at least five times by telephone, and once by post. A psychologist conducted the telephone interview, consisting of the section on anxiety disorders (past year and lifetime), and on depressive disorders (past year only) of the Composite International Diagnostic Interview (CIDI), version 2.1 [22]. The CIDI is a valid and reliable instrument to diagnose psychiatric disorders and can be conducted by trained lay interviewers [23]. If the interview verified the high-risk status (and verified the absence of a fully developed anxiety disorder), a suitable preventive intervention was offered. This could either be assisted self help, internet interventions, a group intervention, or regular treatment for a depressive disorder.

## Results

**Figure 1:**
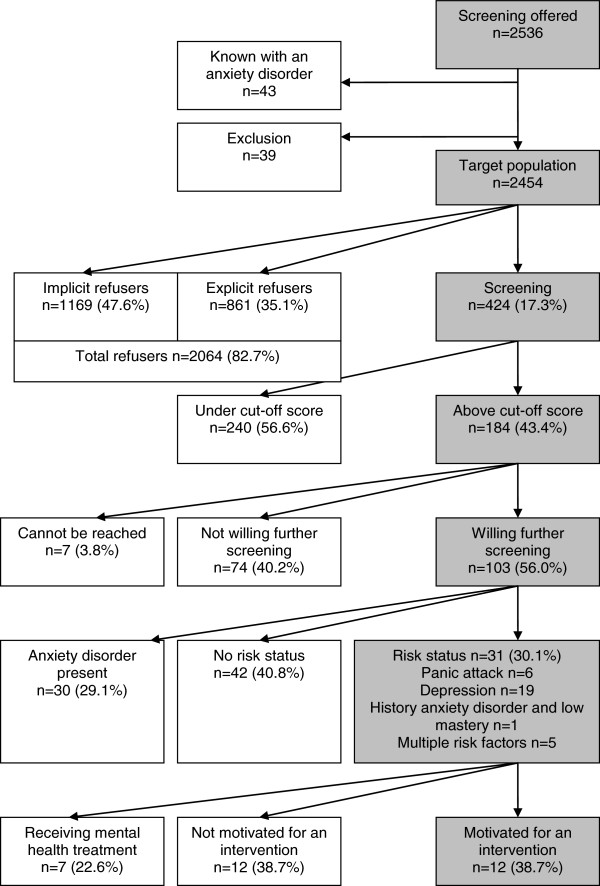
Flowchart screening.

In 2010 and 2011, screening was offered to 2536 individuals visiting their general practitioner, (see Figure 1), 1500 in Amsterdam, 403 in Leiden and 633 in Almere. When screening had been offered to the majority of individuals visiting their general practitioner, screening was ended. Forty-three individuals reported to have been diagnosed with an anxiety disorder. In addition, 39 individuals mistakenly received the package but were excluded later based on age limits or language problems. The target population for screening thus consisted of 2454 individuals.

Of these 2454, 424 individuals (17.3%) completed the questionnaire. This proportion differed significantly between the three general practitioners offices: 14.6% in Amsterdam, 21.5% in Leiden en 21.1% in Almere (*X*^2^ (df = 2) = 18.42; p < 0.001). Of those screened, 63.9% was female, 34.4% male (1.7% missing data), the mean age was 40.5 years (SD 14.0 years)(0.5% missing data); 4.5% only had basic education, 55.9% secondary general education or intermediate vocational education, and 37.8% had higher vocational or scientific education (1.9% missing data).

The other 2030 individuals either explicitly refused to be screened (‘explicit refusers’; n = 861) or did not return the questionnaire (‘implicit refusers’; n = 1169).

Socio-demographic characteristics were provided by 23.4% (n = 468) of all refusers.

Age, gender and educational level did not differ significantly between these refusers and those who completed the screening questionnaire (gender p = 0.31; age p = 0.25; education p = 0.45). In addition, 25.7% (n = 522) provided information about the reason to refuse screening, see Table 2. The most important reasons to refrain from screening were a lack of motivation for preventive interventions (34.9%), emotional burden associated with the presence of a risk status (19.7%), and not considering themselves at risk (17.8%).

**Table 2:** Reasons provided to refuse screening (n = 522)

**Reasons**	**n**	**%**		
No motivation for a preventive intervention	182	34.9		
Emotional burden associated with potential risk status	103	19.7		
Other:	237	45.4	**n**	**%**
Do not consider themselves at risk			93	17.8
Do not find it necessary because they are familiar with anxiety disorders			14	2.7
Objection to provide personal data			4	0.8
Do not feel intervention is necessary until anxiety disorder is present			12	2.3
Logistic objections			17	3.3
Not interested			25	4.8
Not specified			72	13.8

Of the 424 individuals who completed the questionnaire, 43.4% (n = 184) scored positively on the initial screening, and were therefore eligible for further screening by a telephone interview, administered to 56.0% (n = 103). The remaining individuals either refused further screening (40.2%; n = 74) or could not be reached (3.8%; n = 7).

Of the 103 individuals who completed the telephone interview with the CIDI-sections anxiety and depression, 29.1% (n = 30) already fulfilled criteria of an anxiety disorder, and were advised to seek regular treatment. In 40.8% (n = 42) a heightened risk status could not be verified, and in the remaining 30.1% (n = 31) a risk status for the onset of anxiety disorders was present. The most commonly diagnosed target group for prevention consisted of depressive disorders. Of those with a verified risk status, 22.6% already received mental health treatment, 38.7% refused psychiatric treatment despite the verified risk status and a similar proportion expressed willingness for an intervention and was referred (Figure 1).

## Discussion

In theory, the prevention of anxiety disorders appears a good strategy to diminish the burden of disease because anxiety disorders are prevalent and severe, screening on risk status can be conducted safely and effective preventive interventions are available. Moreover, screening for mental disorders has been successfully provided by trained lay interviewers [24]. Such a procedure may limit costs for screening. The importance that individuals attach to prevention, and their willingness to participate in screening and preventive interventions, is unknown. Yet, participation rates in both screening and preventive interventions impact on the disease burden that can be prevented, and on the cost-effectivity of preventive strategies. In the current study the participation rates of screening and preventive interventions with regard to anxiety disorders were assessed by offering screening to 2454 individuals of three general practices, and by offering preventive interventions to those at risk.

We may conclude that the feasibility of the prevention of anxiety disorders in primary care was poor: only a minority of the target population was willing to be screened: 17.3% for initial screening, and of those with a possible risk status 56.0% was motivated for further screening. Of those with a verified risk status, some already received mental health treatment, and some were not motivated to undergo treatment despite the knowledge of being at risk. Thus, offering screening to a large number of individuals resulted in providing preventive interventions in only a few individuals.

To our knowledge, participation rates in screening and interventions aiming to prevent anxiety disorders have not been assessed before. With regard to the present study, several limitations should be taken into account. First, offering screening in only three general practices may have reduced generalizability of the findings; participation rates of screening differed significantly between these practices. However, in none of the practices participation rates were near sufficient to make screening a feasible option. Second, findings cannot be generalized to other preventive programs, because recruitment methods may impact on participation rates. Third, limited data is available about individuals who refrained from screening, because most of them did not return the screening list. As a result, other reasons to refrain from screening than those mentioned above cannot be ruled out.

Despite these limitations, findings of the present study do not stand alone, but correspond with findings regarding the prevention of depressive disorders in which only about 1% of those eligible participated in preventive interventions [25]. Cuijpers and colleagues presented an overview of possible causes of low participation rates [25] (Table 3). Some of the potential causes they describe can be ruled out in the present study: we explicitly reported on risk status thereby excluding unawareness as potential cause, we explained that preventive interventions are effective, mentioned the availability of several preventive interventions, including internet interventions which allow people to follow the intervention at their own pace, and we provided contact addresses to start an intervention of the individuals’ preference. The timing of recruiting participants may have been unsuitable in the present study as potential participants were waiting for their general practitioner to discuss another problem. However, the procedure allowed taking the questionnaire home and returning it later. Given low participation rates when recruiting in the general practitioners office, one could consider other modes of recruitment, such as recruiting participants through systematic screening in the population or via the internet. Because the general practitioner himself was not involved in recruiting participants in the present study, one could also hypothesize that endorsement by general practitioners may improve participation rates, for example, by discussing a potential stigma or by mentioning potential negative consequences of anxiety disorders, including those on cardiovascular function [26]. The two-step procedure might also have discouraged potential candidates. However, this does not seem very likely given that most individuals refrained from screening before taking notice of the procedure. In the present study, emotional burden associated with a potential risk status appeared to be the main reason to refrain from screening. This may well be related to perceived stigma. Alonso and colleagues showed that those with an anxiety disorder frequently feel embarrassed or discriminated related to their mental health [27], and Cuijpers and colleagues [25] suggested stigma as a cause of low participation rates. Other frequently reported reasons to refrain from screening were the assumption not to be at risk of developing an anxiety disorder, and a lack of motivation to use preventive services. It can be hypothesized, that lack of knowledge regarding risk factors and regarding the severity of anxiety disorders may impact upon participation rates. If so, increasing knowledge on anxiety disorders might encourage individuals to participate in screening and prevention.

**Table 3:** **Possible causes of low participation rates in preventive interventions, see**[24]

	
**Participants**	- Individuals do not consider themselves as being at risk.
	- Individuals do not see themselves as having subthreshold complaints because symptoms are labelled differently.
	- Individuals do not believe that preventive interventions are effective.
	- Individuals are not willing to participate because of the stigma associated with mental disorders.
	- Individuals do not want to participate in a group intervention, or have coinciding commitments at the time of the sessions.
**Organization**	- The positioning of preventive services within mental health care may limit the familiarity with these services among potential participants and general practitioners.
**Recruitment**	- Potential participants may not be aware of the existence of preventive services if communication to recruit participants does not reach potential participants.

## Conclusions

Whereas theoretically, screening and prevention of anxiety disorders may substantially reduce the disease burden associated with anxiety disorders, the reduction of the disease burden cannot be capitalized if participation rates in screening and preventive services are low, as was found in the present study. Given the potential benefits, and given the high participation rates in some somatic screening programs such as screening for breast cancer [28], we should not refrain from further attempts to prevent the onset of anxiety disorders. To enable the development of feasible and cost-effective preventive strategies, exploring the reasons of low participation rates, considering involving general practitioners in preventive strategies, and looking at preventive strategies in somatic health care with proven feasibility may be helpful.

## Competing interests

All authors declare that they have no competing interests.

## Authors’ contributions

All authors designed the current study. NB, HM, and BT were involved in data collection. NB performed the statistical analyses. All authors contributed to the interpretation of the results. NB drafted the manuscript. All authors read and approved the final manuscript.

## Pre-publication history

The pre-publication history for this paper can be accessed here:

http://www.biomedcentral.com/1471-244X/12/206/prepub
